# An Atlas of Clinical Microscopy

**Published:** 1886-01

**Authors:** 


**An Atlas of Clinical Microscopy.** By Alexander Peyer, M. D. Translated and edited by Alfred C. Gerard, M. D., Assistant Surgeon U. S. A. First American from the second German edition, with additions. Ninety plates, with 105 illustrations—chromo lithographs. New York: D. Appleton & Co., 1, 3 and 5 Bond street. 1885.

Dr. Gerard is well known and highly esteemed in this city, where, during his short residence, he made many warm friends, and they will join with us in congratulating him upon the excellent work which he has presented to the American profession. To the students and practitioners who own and use microscopes, this volume will be of great service. The plates are most excellent, and the explanatory notes which accompany them are of great value to those not experts in examinations with the microscope.

The book consists of seven parts :

I. Microscopic examination of blood.

II. Microscopic examination of the mammary secretions.

III. Examination of the urine.

IV. Microscopic examination of the sputum.

V. Microscopic examination of the intestinal contents.

VI. Microscopic examination of contents of stomach.

VII. Microscopy of the fluid contents of the various abdominal tumors, of the secretions of the female sexual organs, and the various micro-organisms provoking disease.

An unnecessary amount of space is devoted to "Spermatorrhœa." Plate iii. gives a picture labeled Anthrax Bacilli; but the bacillus is not there. On turning to the text, we find it described as "a *globular* or rod-shaped bacterium!" The text gives Ehrlich's method of demonstration of the only two organisms which, as yet, have been found in human blood during life; but fails to mention that both may be shown by examining the blood in a very thin layer without the addition of any re-agent. (The anthrax bacilli are slender, motionless rods, while the spirilli move very actively.)

After a pretty careful examination of all the plates, this one representation, out of the 105, is the only one we would seriously criticize. The volume is one which should be owned by every physician who uses the microscope, and it will be found an invaluable aid.

